# Concordance between clinical and histopathologic diagnosis and an audit of oral histopathology service at a Nigerian tertiary hospital

**DOI:** 10.11604/pamj.2019.34.100.19388

**Published:** 2019-10-18

**Authors:** Olujide Oladele Soyele, Adetayo Aborisade, Olufunlola Motunrayo Adesina, Abiodun Olatunji, Micheal Adedigba, Adeola Mofoluwake Ladeji, Henry Ademola Adeola

**Affiliations:** 1 Department of Oral Maxillo-facial Surgery and Oral Pathology, Obafemi Awolowo University, Ile-Ife, Nigeria; 2 Department of Oral and Maxillofacial Surgery and Oral Pathology, Obafemi Awolowo University Teaching Hospital Complex, Ile-Ife, Nigeria; 3 Department of Preventive Dentistry, Obafemi Awolowo University Teaching Hospital Complex, Ile-Ife, Nigeria; 4 Department of Oral Pathology and Oral Medicine, Faculty of Dentistry, Lagos State University College of Medicine, Ikeja, Lagos, Nigeria; 5 Department of Oral and Maxillofacial Pathology, Faculty of Dentistry, University of the Western Cape and Tygerberg Hospital, Cape Town, South Africa; 6 Division of Dermatology, Department of Medicine, Faculty of Health Sciences and Groote Schuur Hospital, University of Cape Town, Cape Town South Africa

**Keywords:** Clinical diagnosis, histopathology, descriptive analysis, concordance, clinical audit

## Abstract

**Introduction:**

Although histopathological diagnosis remains the gold standard; good clinical impression is potentially a key diagnostic tool in rural resource-limited settings. Thus, good concordance between clinical impression and histopathological diagnosis is thus a very crucial diagnostic oral pathology tool in low- and middle-income countries (LMICs).

**Methods:**

This retrospective study was performed at the Oral pathology and Oral diagnoses units of Obafemi Awolowo University Teaching Hospital Complex (OAUTHC), Osun state. Clinicopathological reports of all biopsies between the period of 2008-2017 were retrieved and analyzed. Descriptive analysis of data was done using Stata 14. Frequency of oral lesions and rates of accurate clinical diagnoses were evaluated for lesional sites and clinician's qualification/specialization.

**Results:**

In 592 biopsied cases, the mean age was 36.1years with higher female predilection (54.4%). Odontogenic tumors (OTs) were the most prevalent category of lesions (25.3%, n=149), followed by reactive lesions (12%, n=71). Absolute concordance was recorded for 54.6% (k=0.5) of the cases; with highest concordance observed in fibro-osseous lesions (65.6%, k=0.43), and least in pulp/periapical lesions (3.5%). Concordance was higher in females (59.5%, k=0.53) than males (48.3%, k=0.44). Oral medicine specialists had the highest concordance index (62.5%, k=0.59).

**Conclusion:**

The findings in this research indicate that, on a general note, the degree of concordance between clinical and histopathological diagnosis is poor. Hence, improvement in diagnostic skills (irrespective of clinical specialty) is important to improve treatment outcomes, particularly in LMICs. Continuous personnel training and utilization of advanced diagnostic techniques can potentially help bridge the diagnostic gaps.

## Introduction

Patient management in the hospital usually entails a pipeline that commences with a comprehensive investigation of the presenting complaint followed by a thorough examination of various body systems. This enables the clinician to arrive at a provisional diagnosis for the condition with which the patient presented. This art of clinical diagnosis is also supplemented with laboratory investigations in order to validate the provisional impression of the clinician and to finally arrive at a definitive diagnosis. Regardless of the outcome of the physical examination, histopathology and advanced diagnostic techniques remain the gold standard of diagnosis; even though, not all oral lesions warrant further histopathological investigation, as a sizeable number of dental conditions can be diagnosed based on detailed information obtained from clinical features and/ or radiological presentations. Clinical oral examination (COE) remains a principal strategy and valuable means of detecting subtle alterations in the oral hard and soft tissues coupled with clinician's discretion in requesting appropriate investigations for arriving at an accurate diagnosis 1. Despite this, limitations of clinical oral examination in detecting dysplastic lesions, intra-bony lesions, cancers and recurrent mucosal lesions have been fully documented in scientific literature [1-3]. Therefore, a wrong clinical impression might lead to inappropriate investigation; hence, leading to diagnostic pitfalls, delay in management or inappropriate patient management protocols. Varying degree and percentage of discordance between clinical impressions and histopathological diagnoses has been reported in scientific literature, many of which are attributed to defective theoretical knowledge and practical skills in detection of oral lesions [4-7].

In order to avoid the occurrence of misdiagnosis and inappropriate management, American Academy of Oral and Maxillofacial Pathology (AAOMP) have recommended that all “abnormal tissue be submitted promptly for microscopic evaluation and analysis” underscoring the importance of histopathology as a gold standard at reaching a definitive diagnosis [8]. Histological examinations provide information not only on evidence of benign or malignant lesions but clinical behavior of lesions and prognostic information about the lesion, (which might not be achievable with clinical diagnosis) [9,10]. Referrals for biopsy of specimens in a tertiary health centre are not only from specialists (including oral and maxillofacial surgeons, periodontologists, oral medicine specialists, dermatologists and even restorative dentists), but also from general dental practitioners [11,12]. Hence, prompt disease detection and accurate diagnosis is highly essential for effective and appropriate management of pathologies. Due to paucity of documented studies that evaluates the concordance between clinical oral diagnosis and histopathological assessment in our environment, there is an overarching need to identify diagnostic concordance in our African setting, as this would provide further insight into the most efficient management of patients with oral and maxillofacial lesion [5,6]. This study therefore aims at assessing the rate of concordance between clinical and histopathological diagnosis of oral and maxillofacial lesions; and to also conduct an audit of biopsied oral lesions between the period of 2008 to 2017, which is a determinant of oral pathology service utilization; and identification of prevalent lesions in our environment.

## Methods

**Study centre coverage and data capture:** this retrospective study was performed at the Oral Pathology unit of Oral and Maxillofacial surgery and Oral pathology department and Oral Diagnosis unit of the department of Preventive dentistry of Obafemi Awolowo University Teaching Hospital Complex (OAUTHC), Osun State, which is tertiary referral health institution in South-western part of Nigeria (providing diagnostic oral pathology services for neighboring states such as Ondo, Ekiti and parts of Oyo, Delta and Edo. Reports of the clinical information and all biopsies submitted at the Oral Pathology laboratory between the period of 2008 and 2017 were retrieved and analyzed. Data were extracted from clinical record books and histopathology biopsy form using a data collection form (recorded as documented in the histopathology laboratory request form), while histopathology diagnosis was standardized using current terminologies. Demographic data obtained for each case included identification number, age, sex, date and type of biopsy, site of lesion, source of request (department), clinical impression and histopathological diagnoses. These were entered into a database by a single researcher. Exclusion criteria included reports with lack of adequate information, histodiagnostics ambiguity and lesions in which a clinical impression was not given. Repeat (and recurrent) biopsies were recorded singly if the histopathology diagnoses remained unchanged. Lesions were grouped by diagnostic category.

**Definition of concordance:** concordance is recorded when clinical impression corresponds with the definitive histological diagnoses.

**Classification of diagnostic criteria:** researchers have propounded different diagnostic criteria for orofacial lesions in both pediatric and adult populations [7,13-16]. However, due to geographical similarities in population and pattern of disease distribution, a modification of Akinyamoju's diagnostic criteria for classification of oral lesions will be used. This is broadly categorized into a) malignant lesions and b) benign lesions, which is sub-divided into 14 broad diagnostic groups as follows: i. Reactive/ hyperplastic lesions, ii. Cystic lesions, iii. Pulp and periapical lesions, iv. Giant cell lesions, v. Fibro - osseous lesions, vi. Odontogenic tumors (According to 2017 WHO Classification), vii. Epithelial tumours, viii. Mesenchymal tumours ix. Salivary gland diseases, x. Hemato-lymphoid neoplasms, xi. Inflammatory/ microbial diseases, xii. Ulcerative lesions, xiii. Normal tissue xiv. Miscellaneous

**Data analysis:** descriptive statistics carried out were for socio-demographic variables such as age, gender, and location of tumour and prevalence of the lesions were determined. Absolute and relative frequencies were obtained and expressed as percentages. Means and standard deviations were used for continuous variables, while proportions and tables were used for categorical variables. Analysis of each diagnosis entailed: categories of lesions, male: female ratio, age range, mean age and standard deviation. The t-test and ANOVA were used to compare mean differences in the variables; this was after Shapiro-Wilk and Levene test had been used to confirm normality of data and homogeneity of variance respectively. The Chi-square test was used to compare the proportion of the different categories across the age groups and location. Frequency distribution of clinical impressions and histopathological diagnoses was used to describe the concordance rate between the two diagnoses. Sensitivity, specificity as well as positive and negative predictive values were calculated for each lesion: the gold standard being the histopathological diagnosis. 95% confidence intervals were also reported. Cohen's kappa coefficient (κ) between the clinical and histopathologic diagnosis of orofacial lesions was calculated. Cohen's kappa coefficient (κ) is a statistical measure of interrater agreement for categorical items. In general, the following scale was used to calculate concordance using Kappa values: (≤ 0= no agreement, 0.01-0.20= none to slight agreement, 0.21-0.40= fair, 0.41-0.60= moderate, 0.61-0.80= substantial, and 0.81-1.00= almost perfect agreement). All tests were carried out with significance accepted at p < 0.05. The data were analyzed using Stata 14 (Statacorp College Station, Texas, USA).

## Results

A total of 592 biopsied cases were retrieved from the archives of the oral maxillofacial pathology unit between the study period of 2008-2017 at OAUTHC, Osun State. In this period, over 90 differential diagnoses were arrived at from various categories of lesions from clinical examinations: prior to individual samples being sent to the oral histopathology unit. Ameloblastoma (n=114, 19.26%) and pyogenic granuloma (n=56, 9.5%) were the most commonly biopsied lesions followed by squamous cell carcinoma (n=46,7.7%); the 5 most commonly biopsied lesions (ameloblastoma (19.26%, pyogenic granuloma (9.5%), SSC (7.7%), ossifying fibroma (6.42%) and fibrous dysplasia (3.55%)) made up 46.5% of all the biopsied lesion.

**Age and sex:** the mean age of presentation of all biopsied cases was 36.1years ± 18.7 ranging from 2-day old neonate to 94 years with a median age of 33.5 years. 251(42.4%) cases occurred between the third decade (n=141, 23.8%) and fourth decade (n=110, 18.6%) while the least observed is in the extreme of ages together presenting 41, 7% that is; first decade n=29, 4.9%, ninth decade 10, 1.7% and tenth decade 2,0.3%) The percentages of females recorded in this study was 54.4% (n=321) compared to the 45.6% (n=269) of males in a ratio of M: F, 1:1.2. Numerically, females were observed to be higher across all the decades of life except in the eighth decade which had a larger proportion of males and fifth decade with equal predilection. The mean age of presentation for males was 36.1years ± 18.4, which was higher than that of females with 35.9 years ± 18.6 although it was not statistically significant (p>0.05).

**Clinical symptom:** swelling was the most frequent presentation in all biopsy cases, accounting for 478 cases (80.9%). 451 cases (76.3%) were of painless swelling while 27 cases (4.6%) were painful swelling. Symptoms commonly associated with swelling include ulceration (n=26, 4.4%) and purulent discharge (n=24, 4.1%). Ulceration was the second most common presentation with isolated ulcerative cases accounting for 5.8%(n=34). Isolated pain only occurred in 4 cases (0.68%) of all biopsied whilst spontaneous bleeding (n=2,0.34%) was the least documented symptom. The mean age of presentation varies between presenting complaints with painless swelling having a mean age of 33.9years ±18.1, painful swelling 48.2 years ± 18.6, isolated ulcer 45.4 years ± 18.1 whilst swelling associated with ulceration has a mean age presentation of 52.3 years ± 14.7. The differences in mean age is very significant with a p value of 0.001. There are however no significant differences between presenting complaints of the sex (p=0.315), clinical signs and decades of life.

**Duration of lesions:** the mean duration of lesions is 2.5 years ± 3.6 with range from 2 days to 25 years and a median duration of 1 year. The mean duration for males (2.6 years ± 3.9) approximated but slightly higher than that of females (2.5 years ± 3.6) whereas the mean duration per decades varies significantly (p=0.002). Patients within first decade present with a mean duration of 0.9±0.9 which increased progressively to 3years ± 4.9 in the fifth decade and then tailed down to 0.2±3.6 in the tenth decade. The mean duration of presentation with presenting complaints is also very significant (p=0.0002) with mean duration of 2.7years ± 3.6 for painless swelling, 0.8 ± 0.9 for ulcerated lesions, 2.93 years ± 4.7 for painful swelling, 0.9 ± 1.8 for swelling associated with ulceration and 3.9 years ± 4 for swelling associated with purulent discharge.

**Location:** of the 26 documented biopsy sites, mandible was the most common accounting for 275(47.1%) cases. This is followed by the maxilla (n=87,14.9%), the mandibular gingivae (n=49, 8.4%), palate (n=39, 6.7%) and the maxillary gingivae (n=26, 4.4%). the floor of the mouth and other oral mucosa accounted for the least biopsied sites with 5 biopsies each (0.85%) The mean ages of patients regarding location of the biopsy varies and it ranges from 35.2 years ± 16.8 of the mandibular site, 33.6 years ± 18.1 for the maxilla and 45.9 years ± 19.1 for the palate. Others include 23.4years ± 11.6 at the upper lip to 46.2years ± 19.9 at the buccal mucosa with very significant statistical correlation. Regarding gender, there were statistically significant higher female predilection for most of the biopsied locations as greater than 53.5% (maxilla) ± 56.2% (mandible) were observed in the females. This increased significantly to 67.4% and 69.2% in the mandibular and maxillary gingivae respectively and 80% in the lower lip (p=0.01). Higher male preponderance was however observed in the upper lip and tongue (58.3% respectively), floor of the mouth (60%) and palate (69.2%-78.6%). Variations in mean duration were also observed between 0.86 ± 0.7 observed in oral mucosa (excluding buccal mucosa and FOM) and lesions affecting the facial bones (5.8years ± 5.5). The mean duration in respect to most common locations include 2.5 years ± 3.5 (maxilla), 2.9years ± 3.5 (mandible) 1 year for mandibular gingivae and palate and 2.3years ± 5.4 for the maxillary gingivae. This was also highly statistically significant (p=0.0001).

**Figure 1:**
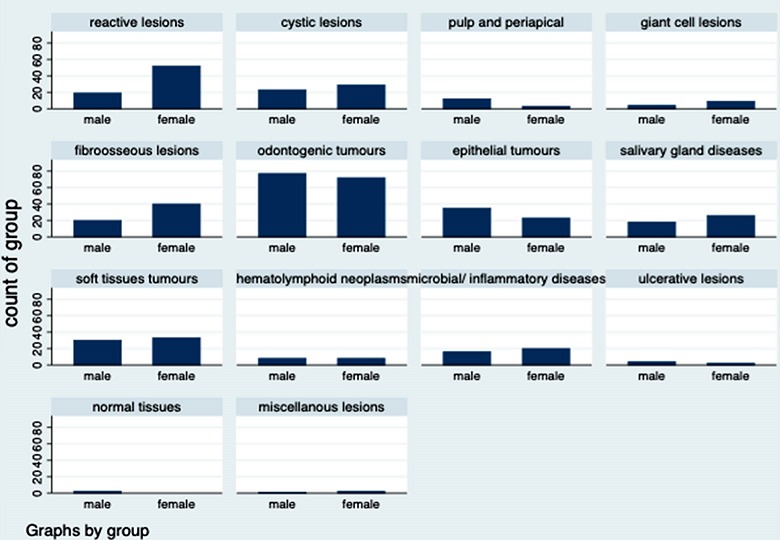
Proportions of categories of lesions per gender

**Categories of lesions:** odontogenic tumors (OTs) were the most commonly observed category of lesions with 25.3% (n=149), followed by reactive hyperplastic lesions which accounted for 12%(n=71) of all the biopsied cases. Biopsy of normal tissue and ulcerative lesions were the least encountered with 2(0.34%) cases and 6(1%) cases, respectively (Table 1). In individual categories, ameloblastoma accounted for 76.5%(n=114) of OTs followed by ameloblastic carcinoma (n=11.4%, n=17) and adenomatoid odontogenic tumors (n=7, 4.7%). Of the reactive hyperplastic lesions (RHLs), pyogenic granuloma (lobular capillary hemangioma) accounted for 56(80%) cases followed by peripheral ossifying fibroma (n=9, 12.9%). Dentigerous cyst (n=20, 38.5%) was the most commonly biopsied cystic lesion followed by odontogenic keratocyst (n=14,26.9%) while the most common biopsied salivary gland lesion was pleomorphic adenoma (n=12, 27.3%). The mean age between the different categories varies significantly (p=0.0001) between 20.7 years +13.4 (haemato-lymphoid lesions) to 52.6years ± 22.1 for epithelial tumors. OTs, RHLs and fibro-osseous lesions (FOLs) presented varying mean ages of 32.3 years ± 12.4, 38.8years ± 21.2 and 29.9 years ± 14.5 respectively. Gender predilection with respect to categories of lesions was found to be very significant (p=0.001), as more female predilection was observed in a greater percentage in these categories, namely; RHLs (73.2%), giant cell lesions (69.2%), FOLs (66.7%), salivary gland diseases (59.1%) and cystic lesions (55.8%) (Figure 1). Male predilection was however observed in pulpal/periapical diseases (80%), ulcerative lesions (66.7%) and epithelial tumors (60.3%).

**Table 1:** Categories of lesions

Group	Frequency	Percent (%)
Reactive lesions	71	12.03
Cystic lesions	52	8.81
Pulp and periapical	15	2.54
Giant cell lesions	13	2.20
Fibro-osseous lesions	61	10.34
Odontogenic tumours	149	25.25
Epithelial tumours	59	10
Salivary gland diseases	44	7.46
Soft tissues tumours	63	10.68
Hemato-lymphoid neoplasms	16	2.71
Microbial/ inflammatory diseases	36	6.10
Ulcerative lesions	6	1.02
Normal tissues	2	0.34
Miscellaneous lesions	3	0.51
**Total**	590	100

**Biologic behavior:** benign lesions accounted for 82.2% (n=486) of all biopsied lesions with mean age of 33.7 years ± 17.6. Female gender was observed to account for 58.6% of all benign lesions and the mean duration is 2.5years ± 3.6. The most common symptom for benign lesions is a painless swelling and most benign lesions occur in the mandible (including gingivae). The mean age of presentation of malignant lesions was 47.5 years ± 19.9, while the male sex accounted for 64.4% of all biopsied malignant lesions. The observed mean duration for malignant lesions was 1.8 years ± 3.2. The most common presentation being ulcerated swelling and favored malignant intra-oral sites are the palate and buccal mucosa. Biologic behavior for sex, mean age, site and mean duration were all statistically significant (p< 0.05).

**Figure 2:**
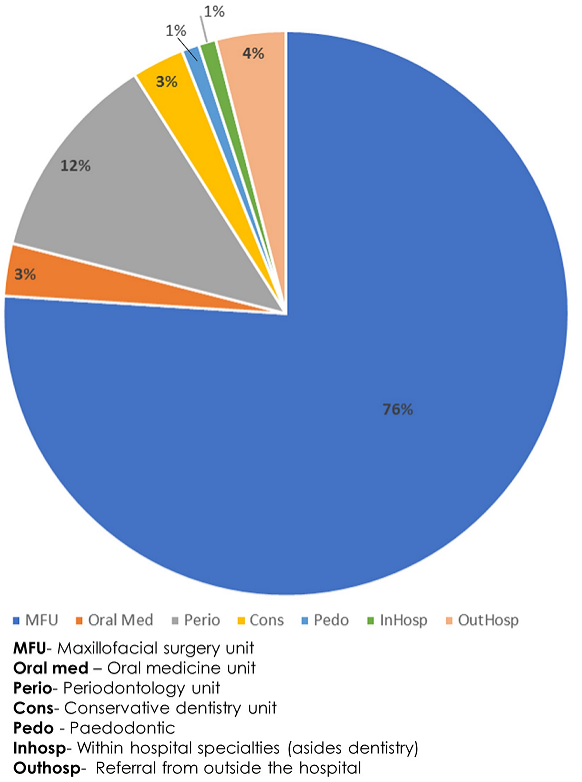
Frequency of source of request

**Source of requisition forms and biopsies:** 95.3%(n=562) of all biopsied cases are from the departments in dentistry with 76.3% (n=450) from oral maxillofacial surgery followed by samples from Periodontology unit (n=71,12%). Biopsy samples from surrounding secondary health institutions and general practice constitutes only 4.2% (n=25) of all cases (Figure 2).

**Diagnostic concordance:** there was 100% concordance between histopathological diagnosis and clinical diagnosis in 54.6% of cases (n=323), whereas in 269 cases (45.4%), there was discordance and hence misdiagnosis. The highest clinico-pathological concordance was found in fibro-osseous lesions (65.6%) and epithelial tumors (66.1%) and least in pulp/periapical lesions (3.5%). Concordance was also observed to be statistically higher with the female gender (59.5%) as against male, 48.3%(p=0.007); and also, significantly higher in benign lesions (57.2%) as compared to malignant lesions. Oral medicine specialists had the highest concordance index at 62.5% whilst outside-hospital specialists (referrals from health centre outside the main tertiary hospital) had the least index at 20% (Table 2). The clinico-pathological agreement as measured by Kappa co-efficient was 0.5 which is rated as Good on the kappa rating scale. The diagnostic concordance in both sexes were rated good even though the kappa rating for the female sex at 0.53 is higher than the male's 0.44. With respect to categories of lesions, the kappa ratings for the common categories are RHLs= 0.11 (very poor), FOLs= 0.43(good), OTs=0.24 (very poor), epithelial tumors=0.4(good) and soft tissue tumors=0.38 (very poor). The maxilla, mandible and mandibular gingivae as sites have good interrater agreement of 0.4, 0.44 and 0.4 respectively whilst the interrater agreement was poor for palate (0.34) and maxillary gingivae (0.32). the kappa's co-efficient for benign lesions was good at 0.52 but malignant lesions' interrater agreement at 0.3 (very poor) Sensitivity, specificity as well as positive and negative predictive values for the selected lesion (ameloblastoma, SSC, pyogenic granuloma, FD and ossifying fibroma) are reported in Table 3 varying from 57.9% for ossifying fibroma to 81% for FD. These suggest that clinical impression has a moderately high probability of correctly detecting lesions that can then be subsequently corroborated by histopathology. Specificity on the other hand ranged from 91.6% (ameloblastoma) to 98.6% (FD); hence implying that clinical impression's capacity to detect healthy tissues is far greater than its capacity to detect disease(s).

**Table 2:** Rate of clinical diagnosis and concordance index among specialties and categories of lesions

Surgeon's specialty	Number	Correct diagnosis	Concordance index	*k*
Oral surgeon	450	259	57.6	0.52
Oral medicine	16	10	62.5	0.59
Periodontist	71	37	52.1	0.22
Conservative specialty	20	7	35	0.05
Out-hospital specialists	25	5	20	0.07
**Gender**				
Male	269	130	48.3	0.44
Female	321	191	59.5	0.53
**Categories of Lesions**				
Reactive lesions	71	43	60.6	
Cystic lesions	52	27	51.9	
Pulp and periapical	15	1	3.5	
Giant cell lesions	13	6	46.2	
FOLs	61	40	65.6	
OTs	149	90	60.4	
Epithelial tumors	59	39	66.1	
Salivary gland diseases	44	15	34.1	
Soft tissue tumors	63	29	46	
Microbial diseases	36	18	50	
Ulcerative lesions	6	6	100	
Normal tissues	2	0	0	
Hematolymphoid neoplasms	16	5	15.6	

**Table 3:** Accuracy of diagnostic tests for most common lesions

Lesions	Prevalence (95%CI)	Sensitivity (95%CI)	Specificity (95%CI)	Positive predictive value	Negative predictive value
Ameloblastoma	0.19(0.16-0.23)	0.68(0.59-0.77)	0.92(0.89-0.94)	0.66(0.57-0.75)	0.92(0.89-0.95)
FD	0.03(0.02-0.06)	0.81(0.58-0.95)	0.99(0.97-0.99)	0.68(0.47-0.85)	0.99(0.98-0.99)
Ossifying fibroma	0.06(0.05-0.09)	0.58(0.41-0.74	0.98(0.96-0.99)	0.61(0.44-0.77)	0.97(0.95-0.98)
SSC	0.08(0.06-0.10)	0.72(0.57-0.84)	0.97(0.95-0.98)	0.64(0.49-0.76)	0.98(0.96-0.99)
Pyogenic granuloma	0.07(0.05-0.09)	0.74(0.58-0.87)	0.97(0.95-0.98)	0.62(0.46-0.76)	0.98(0.97-0.99)

## Discussion

Various publications in scientific literature have reported prevalence of biopsied lesions in entirety [11,13,16-18], groups [14,19,20], categories of lesions [21-23] and amongst anatomic locations [24,25]. Likewise, documented studies have also reported the sensitivity and specificity between clinical impressions and histological diagnosis which is the gold standard of definitive diagnosis [4,5,^26^]. This study aims to report the prevalence of biopsied lesions in our tertiary center and as well as the concordance/agreement rate between clinical impressions and histopathological diagnosis. The total number of biopsied lesions in our study within the period of 2008 and 2017 (10 years) was 592 cases although similar to the prevalence in a documented study [27], it is relatively low in terms of quantity to the number of biopsied lesions recorded in other studies [11,13,28,29]. This may be due to the limited number of dental facilities and few presences of private and/or general dental practices in our coverage areas. This may also be related to the overall reduced dental awareness in our populace and primitive beliefs of the *Hoi- polloi* to adhere to alternative practices and faith-based healing and a general anxiety or fear to any “surgical interventions”. Low utilization of oral biopsies service by dental units such as pediatric dentistry and conservative dentistry could also have contributed immensely to this very low numbers. Unlike in developed western countries where “ALL” excised tissue specimens are referred to the histopathology laboratory, the reverse is usually the case in resource limited centers [1,16].

The mean age of patients recorded in our study was 36.1years ± 18.7 which is similar to the mean age of between 34.9 years and 38 years observed in documented scientific literature [16,28,30] but lower than the mean age of 41years to 54 years observed in other studies [27,31,32]. The peak age of 3^rd^ and 4^th^ decade that was observed in this study also coincides with other studies [16,30]. Gender-wise, a slight female predilection of 54.4% was observed comparable to the reported finding of 51.4% - 59% in other studies [13,27,28,30]. This may be explained in terms of higher positive attitude and oral health service utilization by women compared to male's counterpart in our environment, furthermore males tend to have a poorer health status [13]. However, there are studies with contrasting observation of male preponderances [29,33] while others observed an equal gender prevalence [31]. Difference in gender prevalence of biopsy service may not be unconnected to geographic and epidemiological demographics [16]. Furthermore, this study observed that malignant lesions and odontogenic/non-odontogenic cysts were seen more in males, finding similarly recorded in another study [17]. Similar observation of higher frequency of central(intraosseous) lesions (65%) compared to peripheral lesions in this study was also recorded in the studies of Ali *et al.* [26] and Fierro-Garibay [29]. This is attributed to the high prevalence of OTs and FOLs in the present study which could also be a revelation that most referred lesions for histopathology diagnosis are advanced and aggressive in nature in a resource constrained centre. Other authors have contrary findings with the observation of more peripheral and periapical pathologies of 21.5%, 16.7% and 11.4% in their studies [17,27,30].

Regarding the anatomical site, mandible with 47% was found as the most common site for biopsy, observation that was similarly reported by Akinyamoju *et al.* [16], Moridani *et al.* [30] and Fierro-Garibay *et al.* [31], while maxilla and the gingivae with 14.9% and 12.8% were the next frequent sites in that order. Some researchers in their studies however reported gingivae as the most common biopsied site [34,35]. Although comparison of categories and groups of lesions with previous local and global studies might be difficult which is consequent not only to differences in categorization of diseases, differences in study periods and discrepant study population groups as well as overlaps of some lesions (like radicular cysts as both periapical pathology and odontogenic cyst and also fibromas as reactive lesions or soft tissue tumors) into subgroups and categories that may further complicate subgroup distinctions. Concerning biologic behavior, our study recorded higher frequency of benign lesions with 82.2% while malignant lesions accounted for 17.8%, this is comparable to 74.4% and 25.6% of benign and malignant lesions observed in Akinyamoju *et al.* [16] study but significantly lower than other studies in documented literature [28,30]. Contrary to the documented reports in the scientific literature where reactive lesions and periapical pathology accounted for the most biopsied category of lesions [17,27,28], our study found OTs as the most frequently biopsied category of lesion (25.2%) followed by reactive lesions constituting 12% of all biopsied lesions. High utilization of biopsy services by Oral and Maxillofacial surgery specialty and high incidence of Ameloblastoma among black Africans could probably explained the high frequency of presentation of OTs for biopsy in our center. Furthermore, in this study, Maxillofacial surgery contributed 76.3% of all biopsied cases followed by 12% from periodontology and 4.2% from adjoining primary and secondary health care centers. This similar trend is observed in the studies conducted in Iran [5,30], Spain [31] and among Nigerians [16]. This is however at variance with studies of Jones *et al.* [17], Bhaskar *et al.* [18] and Raquel Sixto-Requeijo [27], where private practitioners, general dental practices and oral medicine specialty accounted for a larger percentage of their referrals respectively. This observation corroborates the low utilization from other dentistry units in our centre. Ameloblastoma was the most commonly diagnosed lesion (19.5%) in this study concurring with the study of Akinyamoju *et al.* [16] but in sharp contrast to documented reports elsewhere, where fibrous epulis [36], inflammatory fibrous hyperplasia [34], fibroma [27], odontogenic cysts [30] were the most common lesions.

Pyogenic granuloma was the most frequently diagnosed reactive lesion in our study, this was similarly observed in the study by Raquel Sixto-Requeijo *et al.* [27] and at variance with fibrous hyperplasia by Fierro-Garibay [31] and inflammatory fibrous hyperplasia by Takashima *et al.* [34]. Other most common biopsied lesion reported in multifarious studies were radicular cyst, periapical granulomas and other mucosal pathologies [17,30,34]. These studies were conducted in centers with robust private and general dental practice(s), and where all excised tissue (including normal tissue) were sent for histopathologic examination. The most frequently diagnosed epithelial tumor and the most common biopsied malignant lesion in this study was oral squamous cell carcinoma; hemato-lymphoid malignancies was however the most common in the study by Ali *et al.* [28]. In our study, oral squamous cell carcinoma accounted for 0.1% of all the biopsied lesions which is similar to the range of 0.1 - 0.4% documented in other studies [17,31]. Factors, such as age, gender, socioeconomic status, prosthesis use, smoking and alcohol consumption have been demonstrated to be associated with incidence of oral lesions [37] but these factors (asides age and gender) are not routinely imputed or requested in biopsy requisition forms, hence corroborating their significance and associations either singly or cumulative is beyond the scope of this study The significance of an accurate clinical impression cannot be overemphasized as it guides the investigative modalities and definitive treatment for patients. Menedez *et al.* [4], in their study correlated concordance with diagnostic categories and this was achieved when the clinical impression and histological diagnoses belonged to the same diagnostic category. This will not only create a systematic bias as it increases the proportion of concordance but also underlie the fact that lesions in same diagnostic category presents with dissimilar clinico-radiological presentation i.e. malignant ameloblastoma and squamous odontogenic tumor. Thus in our study as similar to study in documented scientific literature [7], we cite concordance when clinical impression is the same as histological diagnosis. Discordance between this is termed a misdiagnosis. Concordance was measured by concordance index and by interrater agreement (of Cohen's) hence an overall concordance index of 54.6 was observed in our study which was comparable to the 50.6-56.4 recorded in the studies of Patel *et al.* [38] and Williams *et al.* [39]. Our concordance index of 54.6 was however low when compared to studies in documented scientific literature of 69.1 value by Azmoodeh *et al.* [26], 66.6 by Seifi *et al.* [40], 64.5 by Emeka *et al.* [7], and 80.7 observed by Fattahi *et al.* [41]. This can be accrued to a more stringent measure employed in our study as against some studies [4,7], where the concordance was flexible, coupled with accommodating concordances within similar categories or group of lesions; unlike in our study where lesions were exact. In terms of correlation of gender to concordance, we observed a higher concordance index in females (59.5) as against males (48.3), with kappa's co-efficient of 0.53 and 0.44, respectively. This is at variance with Tatli *et al.* [6], who reported a slightly higher male concordance (0.92) compared to females (0.9). The higher female concordance in this study may be attributed to accurate account of related details and leading information of lesions by female patients. In other studies [7,42], it was opined that females tend to present earlier at the onset of the lesion, thereby making the diagnosis relatively easier.

Regarding age, highest concordance was observed within the 7^th^ decade and above with concordance index of 70; this was in tandem with reports in documented literature [41,43,44]. It has been proposed that reduced number of lesions in this age bracket (after exclusion of lesions that develop in children and young adults), limited number of newly developing lesions and loss of teeth at these decades might account for the high concordance observed [41]. Significantly, variable concordance was also observed with specialties, with the highest concordance index (62.5) emanating from oral medicine specialists, followed by oral maxillofacial surgeons (57.6) and periodontologists (52.1). When compared with oral medicine specialist, the vast array of oral maxillofacial lesions presented to the oral surgeons and the profound similarities between hyperplastic lesions and periodontal diseases could have contributed to the reduced concordance indices of the oral surgeons and periodontologists, respectively. Seifi *et al.* [40], in their study however recorded the highest concordance amongst oral surgeons. The overall interrater agreement was 0.5 which was slightly higher than the previous Nigerian study by Emeka *et al.* [7] (0.45), but lower than 0.61 reported by Seifi *et al.* [5]. Intraoral sites with the highest concordance index and interrater agreement were the tongue, lower lip, buccal mucosa and mandible, respectively. This is comparable to the observations of Emeka *et al.* [7] and Tatli *et al.* [6] where the tongue, buccal mucosa and salivary glands had the highest concordance. Tatli *et al.* [6] attributed this to the specific characteristics of lesions in these sites. In general, concordance rates between the pathologists, surgeons and other specialties can be improved by ensuring proficiency and good interdisciplinary relationship, mostly between pathologists and surgeons (including other specialties). Accuracy of the interpretation of plain radiographs and imaging techniques; good and adequate biopsy specimen, coupled with appropriate means of conveying specimen to the laboratory, would also improve concordance [41]. Discordances should be meticulously examined by all specialties (particularly oral pathology), to improve early detection and diagnosis of diseases and biopsied lesions [6].

## Conclusion

Clinical diagnosis remains the foremost diagnostic instrument after detailed patients' anamnesis and clinical oral examination. A good clinical acumen may also be the only available diagnostic tool in resource limited centers and primary health care facilities. Hence, its capacity and prognostic potential as a sensitive and specific diagnostic instrument cannot be overemphasized. This guards against misdiagnosis and inappropriate treatment. Age, gender and anatomic sites are pertinent demographic characteristics that can be utilized for the differential diagnosis of major oral lesions. Given the observed low to moderate sensitivity of clinical diagnosis of oral lesions, all dental specialties and sub-specialties should be advised on the need to send all excised specimens for histopathological analysis and diagnosis to reduce misdiagnosis and by extension inappropriate treatment. Multidisciplinary, clinico-pathological meetings and seminars should also be encouraged at dental centers to minimize diagnostic discordance rates between different dental specialties.

### What is known about this topic

- Clinical oral examinations and investigation is the foundation for good clinical impression and appropriate individualized therapy;
- Although histopathological diagnosis remains the gold standard; good clinical impression is potentially a key diagnostic in rural resource-limited settings;
- There has been a huge variation between clinical impression and histopathological diagnosis.

### What this study adds

- Improvement in diagnostic acumen (irrespective of clinical specialty) is important to improve treatment outcomes, particularly in low- and middle-income countries (LMICs);
- Good concordance between clinical impression and histopathological diagnosis is a crucial diagnostic oral pathology tool in LMICs;
- High index of concordance between oral pathology and other specialties with continuous personnel training and utilization of advanced diagnostic techniques can potentially help bridge the diagnostic gaps.

## Competing interests

The authors declare no competing interests.
